# The knowledge and attitudes of South African-based runners regarding the use of analgesics during training and competition

**DOI:** 10.17159/2078-516X/2022/v34i1a13976

**Published:** 2022-01-01

**Authors:** R Thorpe, M Blockman, H Talberg, T Burgess

**Affiliations:** 1 Division of Physiotherapy, Department of Health and Rehabilitation Sciences, University of Cape Town, South Africa; 2 Division of Clinical Pharmacology, Department of Internal Medicine, University of Cape Town, South Africa

**Keywords:** athletes, NSAIDs, performance, exercise

## Abstract

**Background:**

The use of analgesics is prevalent in runners, with the associated potential for serious harm. However, there is limited information regarding runners’ knowledge and attitudes towards the use of analgesics in relation to running.

**Objectives:**

To describe South African-based runners’ knowledge and attitudes regarding running-related analgesic use.

**Methods:**

This study has a descriptive, cross-sectional design. South African-based runners, over the age of 18 who ran at least one race in the year preceding the study were included in this study. Participants completed an online questionnaire, including sections on demographic information, training and competition history, pain medication use, and knowledge and attitudes regarding running-related analgesic use.

**Results:**

Data from 332 participants were analysed. Attitudes regarding the use of analgesics in relation to running were generally positive; however, knowledge was poor, with only 20% of participants achieving adequate knowledge scores (75% or above). Very few (n=49; 15%) had both adequate knowledge and positive attitudes, with most respondents (n=188; 58%) having inadequate knowledge and negative attitudes. Negative attitudes towards the use of analgesics were found to increase the odds of running-related analgesic use (OR 2.32; 95% CI:1.31–4.11).

**Conclusion:**

Knowledge regarding running-related use of analgesics was inadequate. Despite a lack of knowledge, attitudes were positive. Participants displayed positive attitudes towards safe practice regarding running-related analgesic use, but these did not translate into good practice. Targeted interventions are required to educate runners and improve their knowledge of all the effects associated with running-related analgesic use.

Global participation in running has continued to grow over the last decade with millions of people running weekly.^[1,2]^ This growth has been observed across all distance categories from the social 5km ParkRun to 100-mile (160,9km) ultramarathons.^[1,2]^ In 2016, nine million runners finished races globally.^[3]^ Of particular interest to sports researchers involved in training/distance and load and its link to injury is participation in long-distance running events, such as half marathons, marathons, and ultramarathons. These distance events require months of progressive training and load adjustment, which, in combination with multiple other variables, like body mass index (BMI), age, sex, pace and previous injury, may all increase the risk of the participant developing a running-related injury (RRI).^[4]^ A RRI can be a source of immense psychological and psychosocial stress for runners,^[5]^ especially when considering the physical, mental, and social benefits of running.^[6]^ Runners therefore tend to engage in various untested and ill-advised practices to continue running despite injury. The use of analgesics, including non-steroidal anti-inflammatory drugs (NSAIDs), is one option that runners may consider to facilitate continued participation in running. Adoption of this as an injury mitigation strategy is seen in the high and rising use of analgesics in running, and sport in general.^[7,8]^ Major concerns surrounding increased analgesic use in sports is the likelihood that this practice is unsupervised and not supported by adequate knowledge of the effects of drugs and their side effects.^[9]^ Potential adverse effects from NSAIDs consumption during running include increased physiological and systemic stresses with the consumption of NSAIDs during 10km and 21.2km races which have been shown to increase urinary neutrophil gelatinase-associated lipocalin (uNGAL), an indicator of acute kidney injury.^[10]^

Previously reported rationales for the use of analgesics in sport include perceived improvements in performance, and prophylactic injury management;^[11]^ which are unsupported by scientific evidence; and further questions athletes’ knowledge of and attitudes towards the use of analgesics.^[12]^ When combined with the ease of over-the-counter (OTC) access in South Africa to complex analgesics, such as multi-ingredient NSAIDs, this is a major cause for concern in endurance sport,^[7,11]^ including running, where there has been limited research to date.

Our study aimed to determine and describe the knowledge and attitudes regarding the use of analgesics in South African-based runners.

## Methods

### Study design and ethical approval

The methods for this study have previously been described in full,^[7]^ and will be summarised to avoid replication.

This study obtained ethical approval from the Faculty of Health Sciences Human Research Ethics Committee, University of Cape Town (HREC REF: 093/2016) and as mentioned, had a descriptive cross-sectional design.

### Participants

Recruitment for the online survey was done via South African running clubs and social media platforms. Inclusion in the study required that participants be South African-based runners of at least 18 years of age, with internet access, who ran at least one race of any distance, per year. A race was regarded as a competitive event that was open to any runners and external to a single running club’s calendar. Runners were excluded if informed consent was not provided, or they failed to complete the knowledge section of the survey.

### Sample size determination

Sample size was calculated based on data from previous studies that determined prevalence rates of prescribed and over-the-counter NSAID use in athletes^[8,11]^, where the required sample size was 208 participants.

### Measurement instrumentation: Questionnaire

An investigator developed and an expert validated the questionnaire created to determine participants’ knowledge and attitudes regarding the use of analgesics in running, as described previously.^[7]^

Knowledge was scored by awarding participants one mark for each correct answer and zero marks for incorrect answers. Participants were graded using the percentage of correct answers, i.e. 75% and above demonstrated adequate knowledge and below 75% demonstrated inadequate knowledge. This scoring system was based on previous research.^[13]^ Attitudes towards analgesics were determined by using a five-point Likert Scale. Several statements were provided and participants needed to indicate the extent to which they agreed with each statement, ranging from ‘strongly agree’ to ‘strongly disagree’. They were also asked to state the likelihood that they would use analgesics in specific situations, ranging from ‘very unlikely’ to ‘very likely’. Each point on the Likert-scale was weighted, with one point being awarded for the ‘most negative’ response and five points being awarded for the ‘most positive’ response. A positive response was one that was seen to promote and be aligned with safe or healthy behaviours regarding analgesics and their use in running, whereas a negative response was seen to be aligned to potentially unsafe behaviours. Percentages of 75% and above indicated positive attitudes and below 75% indicated negative attitudes.

The knowledge and attitudes sections of the questionnaire can be found in Appendix A.

### Procedure

The final questionnaire was uploaded to the online survey website SurveyMonkey® (www.surveymonkey.com) and was open for one month. It was the intention of the researchers to translate the questionnaire into Afrikaans, IsiXhosa and Isizulu. However, due to the rapid rate of responses, the study was closed to further enrolment earlier than predicted and these translations were not included in the final study.

### Statistical analysis

Statistical analyses were performed using the IBM SPSS software (IBM Corp. 2015. IBM SPSS Statistics for Windows, Version 23.0. Armonk, NY. www.ibm.com). A Shapiro-Wilk test was used to determine whether the data were normally distributed. Frequency tables and Pearsons’ Chi-squared measures of association were used for categorical variables. Odds ratios and 95% confidence intervals (95% CIs) were calculated using VassarStats (http://www.vassarstats.net/odds2x2.html) to determine associations between individual variables and analgesic use.

## Results

### Participants

**Fig. 1:**
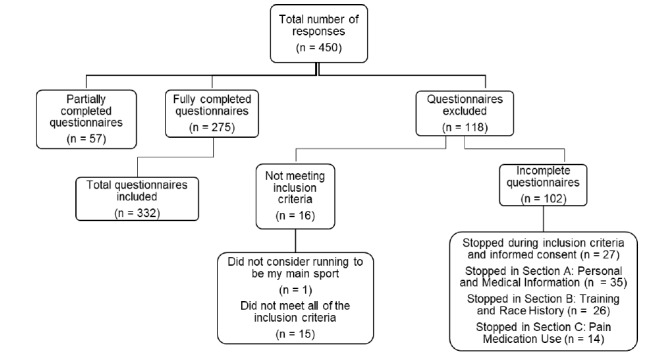
Summary of study respondents

We received 450 responses; 16 responses were excluded as they failed to meet the inclusion criteria. During data analysis, a further 102 responses were excluded as participants failed to complete all the mandatory sections of the questionnaire. Overall, 275 fully completed survey responses and 57 partially completed responses were included in the study. Partially completed responses were questionnaires that were completed up until the end of the knowledge section of the questionnaire but where the final attitudes section of the questionnaire was incomplete. Therefore, data from 332 participants, 196 (59%) females and 136 (41%) males, were included (Figure 1). Participant age ranges and training history are seen in Table 1.

### Knowledge and attitudes regarding the use of analgesics

Fewer than 20% (n=65) of participants demonstrated adequate knowledge regarding analgesic use. However, 73% (n=237) demonstrated positive attitudes towards the use of analgesics. Only 49 participants (15%) showed both adequate knowledge and positive attitudes (Table 2).

Regarding the participants’ specific responses, only 53% could correctly identify the possible side effects of NSAIDs and only 21 participants (6%) were correctly informed as to the most suitable time to take NSAIDs. Less than 50% knew that aspirin was both an analgesic and NSAID, while only 11% were aware that intra-articular corticosteroids have less adverse effects than the oral effects. Although almost two-thirds of participants (63%) correctly indicated that topically administered NSAIDs had fewer adverse effects than orally administered NSAIDs, only 27% were aware that it is not recommended to use oral and topical NSAIDs concurrently (Table 3).

Participants demonstrated positive attitudes regarding the use of analgesics. A total of 110 participants (33%) disagreed and 85 (26%) strongly disagreed with the statement that the prophylactic use of analgesics before a run will prevent pain during a run, while 57% and 42% of participants strongly disagreed that oral analgesics or topical analgesics are an important part of their running preparations, respectively. More than 100 participants (32%) agreed that they would only use analgesics when running if they were injured, and 33% agreed that they would use analgesics specifically for a race if they were injured. Analysis of the question whether analgesics are not seen to have an important role in running, showed that 122 participants (37%) strongly disagreed with this statement, and 265 participants (80%) feel that runners are not sufficiently educated regarding the effects and side-effects of analgesics (Table 4).

There was a significant difference between the combined knowledge and attitudes scores (χ^2^ = 9.64; p = 0.022) in participants who used analgesics in running and those who did not, yet there were no significant differences in knowledge scores between these groups. There were no significant differences in the knowledge and attitude scores found between participants that made use of multiple analgesics concurrently and those who only used one type of analgesic (Table 5).

Negative attitudes towards the use of analgesics were found to increase the odds of running-related analgesic use (OR 2.32; 95% CI: 1.31–4.11) when compared to positive attitudes.

## Discussion

### Knowledge Scores

This is the first study that we are aware of that specifically grades participants’ knowledge regarding analgesic use, especially in running. Scores of 75% and above for the knowledge and attitudes sections of the questionnaire were classified as adequate.^[13]^ Participants’ overall knowledge regarding analgesics was inadequate, with less than 20% of participants scoring 75% or above.

Participants generally displayed good knowledge regarding the general risks of analgesic misuse and overdose, similar to what has previously been found in distance runners.^[14]^ Yet, they scored poorly on the questions concerning adverse effects, drug interactions, and the effects of specific analgesics, especially NSAIDs. These findings are supported by previous literature in both the general and sporting populations.^[8,15]^ There is an awareness that there are risks when using NSAIDs, however, there is a lack of awareness regarding the specifics of the adverse effects. This lack of knowledge could present with significant implications as runners may not associate specific adverse effects to the use of NSAIDs. For example, a runner may experience abdominal pain as an adverse effect of NSAID use; yet, as they are unaware that the associated abdominal pain is NSAID induced, they consume further NSAIDs, or alternative analgesics, to manage this abdominal pain, placing them at higher risk of more serious adverse effects due to the cumulative dose or potential drug interactions. Runners need also be aware of the exercise-induced stress on physiologic function because of distance running, and the further detrimental effects that NSAID consumption can have on these systems. These changes are specifically seen in the renal function of distance runners when uNGAL, an indicator of acute injury and tubular dysfunction, is measured. Running a 21.1km race caused a significant increase in uNGAL that this is further increased if NSAIDs were consumed, showing a greater risk of kidney damage in runners.^[10]^

### Attitude scores

Despite previous studies identifying that athletes generally demonstrate negative attitudes towards the use of analgesics,^[8,11]^ the participants in our study had positive attitudes towards analgesic use, with 73% achieving adequate attitude scores. Negative attitudes towards the use of analgesics increase the odds of running-related analgesic use. Attitudes have been shown to be a variable predictor of health behaviours, and have previously been predictive of alcohol and marijuana use but were not predictive of positive or negative smoking behaviours.^[16]^ In our study, 143 participants (68%) used analgesics in running despite having positive attitudes towards their use. This behaviour could link to the stereotype that athletes will do anything to achieve the best results in their sport, striving for success at all costs; even though they are aware that there are potential health risks.^[17]^ It has previously been seen that 33% of runners using analgesics, used these to aid recovery from a running injury and facilitate continued participation, showing that runners participate in high-risk behaviours for their sport.^[7]^ This behaviour could also be related to the influence of the community around the runner and how they affect and shape attitudes and behaviours.^[18]^ It is further supported by the data that membership of a sports club is predictive of self-medication. ^[19]^ These are important considerations when attempting to promote safe analgesic practice in runners.

Eighty percent of participants disagreed with the statement that runners are sufficiently educated regarding the effects and side-effects of analgesics. This is a very important finding as it highlights the need for further input and education of runners, coaches, and running clubs regarding safe and appropriate analgesic use.

Inconsistency existed in the combined knowledge and attitudes scores of participants as most participants scored poorly in the knowledge section of the questionnaire but well in the attitudes section. This is an interesting finding as participants’ knowledge regarding analgesic use could be expected to influence their attitudes towards use, which was not the case.

### Limitations and recommendations

As the questionnaire was only available in English, it may have reduced the generalisability of the results of our study to a wider population. The fact that the questionnaire was only available online is a further limitation as the participants may not be representative of a lower socioeconomic group that may display different knowledge and attitudes to the participants in our study. Their potential lack of participation, would more likely be due to the high mobile data costs in South Africa, that runners from a lower socioeconomic grouping may not be able to afford, rather than a lack of access to the internet.^[20]^ As the study questionnaire relied solely on self-reported data, which could not be independently verified, this may have biased the results due to recall bias.

The ways that runners are educated regarding the safe use of analgesics, NSAIDs specifically, should be further investigated to determine the best means to address the high usage of NSAIDS, including combination analgesics, highlighted by our findings. The roles of social media, running clubs, and various media sources should be explored as the need for a formal education campaign amongst runners is evident.

## Conclusion

Our study looked at the specific knowledge and attitudes of runners towards analgesic use and has revealed important gaps in the specific knowledge and attitudes of runners towards analgesic use. Despite our participants displaying positive attitudes towards the use of analgesics in relation to running, they had inadequate knowledge. Hence these positive attitudes did not translate into safe practice as can be seen by the numbers of analgesic users and their patterns of use to continue participation despite pain or injury. The high usage of analgesics together with inadequate knowledge of their potential adverse effects increases the likelihood of severe complications during training and competition.

This study highlights the urgent need to educate runners about the negative effects of analgesic use, either before, after or during training and competition. All stakeholders involved in providing information around analgesic use, both officially and unofficially, as well as pharmacists who dispense OTC analgesia should be targeted in an education campaign to improve runners’ analgesic knowledge and their safe use in training and competition.

## Supplementary Information



## Figures and Tables

**Table 1:** Age ranges, and training and competition history of male and female respondents (n = 332)

	Male (n=136)	Female (n=196)	Total (n=332)
**Age (years)**	39 ± 10	38 ± 10	38 ± 10

**Number of years of running**			
0 to 3 years	35 (26%)	62 (32%)	97 (29%)
4 to 9 years	47 (35%)	78 (40%)	125 (38%)
10 or more years	54 (40%)	56 (29%)	110 (33%)

**Kilometres run per week**			
39 km or less	70 (52%)	121 (62%)	191 (58%)
40 km or more	66 (49%)	75 (38%)	141 (43%)

**Marathon or Ultra-Marathon completion**			
Yes	75 (55%)	96 (49%)	171 (52%)
No	61 (45%)	100 (51%)	161 (48%)

**Number of Marathons or Ultra-Marathons per participant**			
Marathons	3 ± 2	2 ± 2	3 ± 2
Ultra-marathons	2 ± 2	2 ± 1	2 ± 1

Data are expressed as number of responses (n) and column percentages (%) or mean ± standard deviation (SD)

**Table 2:** Knowledge and attitudes scores regarding the use of analgesics

	Participants (n=332)
**Knowledge scores**	
Inadequate knowledge (<75%)	267 (80%)
Adequate knowledge (>75%)	65 (20%)

**Attitudes Scores**	
Negative attitudes (<75%)	86 (27%)
Positive attitudes (>75%)	237 (73%)

**Combined Knowledge (K) and Attitude (A) Scores**	
Poor K and A	70 (22%)
Poor K Good A	188 (58%)
Good K Poor A	16 (5%)
Good K and A	49 (15%)

Data are expressed as number of responses (n) and column percentages (%). Column n values of <332 for participants are as a result of partially completed questionnaires.

**Table 3:** True and false question responses (n = 332)

Question	False	True	I don’t know
It is safe to take over-the-counter pain medication if you have been drinking alcohol	**305 (92%)**	7 (2%)	20 (6%)
If the recommended dose of pain medication doesn't relieve your pain, it is safe to take more	**312 (94%)**	4 (1%)	16 (5%)
Local anaesthetic injections can cause heart problems	29 (9%)	**112 (34%)**	191 (58%)
Aspirin can be both an analgesic and an anti- inflammatory	53 (16%)	**151 (46%)**	128 (39%)
Panado® is a stronger pain medication than Codeine	**247 (74%)**	7 (2%)	78 (24%)
Injected corticosteroids (Cortisone) are safer than oral/tablet corticosteroids (Cortisone)	95 (29%)	**35 (11%)**	202 (61%)
It is possible to overdose on Panado® (Paracetamol)	23 (7%)	**256 (77%)**	53 (16%)
Anti-depressant medication can be used to manage pain	135 (41%)	**50 (15%)**	147 (44%)
Paracetamol and anti-inflammatories work in the same way	**223 (67%)**	18 (5%)	91 (27%)
Topical pain medication (gels and patches) have fewer side effects than other forms of pain medication	45 (14%)	**210 (63%)**	77 (23%)
It is safe to use oral (tablets) and topical anti- inflammatories at the same time	**90 (27%)**	95 (29%)	147 (44%)
All types of topical pain medication have the same side effects	**211 (64%)**	13 (4%)	108 (32%)
It is safer to use topical pain medication, rather than oral pain medication, if you are using other types of medication (i.e. diabetic or cholesterol medication)	40 (12%)	**129 (39%)**	163 (49%)

Data are expressed as number of responses (n) and percentages of respondents (%). The correct responses are in bold.

**Table 4:** Attitudes towards analgesics in running (n = 332)

Statement	Strongly agree	Agree	Neutral	Disagree	Strongly disagree
Taking pain medication before a run will stop me from feeling pain during the run	5 (2%)	64 (19%)	59 (18%)	110 (33%)	85 (26%)
Taking pain medication before a run will stop me from feeling pain or stiffness after the run	3 (1%)	22 (7%)	57 (15%)	156 (47%)	91 (27%)
Oral pain medication (Paracetamol/anti- inflammatories) is an important part of my running preparations	5 (2%)	13 (4%)	27 (8%)	88 (27%)	190 (57%)
I would use pain medication for training: If I had pain (an injury)	13 (4%)	85 (26%)	35 (11%)	78 (24%)	112 (34%)
I would use pain medication for a race: If I had pain (an injury)	17 (5%)	110 (33%)	36 (11%)	55 (17%)	105 (32%)
I would use pain medication as part of my recovery	13 (4%)	115 (35%)	56 (17%)	67 (20%)	72 (22%)
I would only use pain medication when running if I was injured	13 (4%)	105 (32%)	42 (13%)	81 (24%)	82 (25%)
Using pain medication before training or a race will improve performance	2 (1%)	19 (6%)	39 (12%)	104 (31%)	159 (48%)
Using pain medication will speed up recovery	4 (1%)	45 (14%)	63 (19%)	103 (31%)	108 (33%)
Pain medication has an important role in running	7 (2%)	30 (9%)	58 (18%)	106 (32%)	122 (37%)
Runners are educated enough with regards to the effects and side-effects of pain medication	4 (1%)	15 (5%)	39 (12%)	130 (39%)	135 (41%)

Data are expressed as number of responses (n) and percentages of respondents (%). Row n values of <332 for participants are as a result of partially completed questionnaires.

**Table 5:** Knowledge and attitudes towards running-related analgesic use.

	Analgesic use in running			Χ^2^	p	Odds Ratio (95% CI)
	No (n=120)	Yes (n=212)	Total (n=332)			
**Knowledge scores**						
Inadequate knowledge (<75)	97 (81%)	170 (80%)	267 (80%)	0.02	0.89	1.04 (0.59 – 1.84)
Adequate knowledge (>75)	23 (19%)	42 (20%)	65 (20%)			

**Attitude Scores**						
Negative attitude (<75)	19 (17%)	67 (32%)	86 (27%)	8.56	0.003**	2.3 (1.31 – 4.11)**
Positive attitude (>75)	94 (83%)	143 (68%)	237 (73%)			

**Combined knowledge (K) and attitude (A) scores**						
Inadequate K and Negative A	17 (15%)	53 (25%)	70 (22%)	9.64	0.022*	
Inadequate K and Positive A	73 (65%)	115 (55%)	188 (58%)			
Adequate K and Negative A	2 (2%)	14 (7%)	16 (5%)			
Adequate K and Positive A	21 (19%)	28 (13%)	49 (15%)			

Data are expressed as number (n) and percentage of respondents (%). CI, Confidence Intervals;

** indicates p<0.01;

* indicates p<0.05.

Total column n values of <332 for participants are as a result of partially completed questionnaires.
