# Supplementary material for: The knowledge and attitudes of South African-based runners regarding the use of analgesics during training and competition
Source: S Afr J Sports Med. 2022 Jan 1;34(1):v34i1a13976. doi: 10.17159/2078-516X/2022/v34i1a13976 (PMC9924556; doi:10.17159/2078-516X/2022/v34i1a13976)
Supplement: Supplementary file 1 [file 2078-516X-34-v34i1a13976-s001.pdf]

The knowledge and attitudes of South African-based runners regarding the use of analgesics during training and competition

Appendix A: Knowledge and Attitudes Sections

Knowledge and Attitudes Towards Pain Medication
**The following section is divided into two subsections:**<br>**Section 1) will ask questions related to your knowledge of pain alleviating medication.**<br>**Section 2) will ask questions specifically related to your attitudes towards the use of pain alleviating medication in running.**
1) Knowledge of Pain Medication
The following section refers to various types of pain medication, such as:<br>***Paracetamol (Panado®)***<br>***Non-Steroidal Anti-inflammatories (NSAIDs) (Myprodol®, Cataflam®, Brufen®, Aspirin, etc.)***<br>***Muscle Relaxants (Spasmed, Valium®)***<br>***Cortisone (Prednisone®, Celestone®)***<br>***Codeine***<br><br>***These are just a few examples of the various types to assist you in the following section.***
Please choose only ONE answer for each of the questions in section 1)

Knowledge and Attitudes<br>1) Knowledge of Pain Medication	
Which of the following are side effects of anti-inflammatories?	Gastrointestinal/stomach problems (e.g. stomach cramps, ulcers, nausea)<br>Cardiovascular/heart problems (e.g. heart attack, chest pains, stroke)<br>Kidney problems (e.g. kidney failure, blood in urine)<br>Options A, B & C<br>Options A & B only
Which of the following is NOT an anti-inflammatory?	Aspirin<br>Cataflam®<br>Brufen®<br>Voltaren®<br>Panado®
When is the best time to take anti-inflammatories?	On an empty stomach<br>10-20 min before a meal with some milk or yoghurt<br>After a meal<br>First thing in the morning<br>At any time
It is safe to take anti-inflammatories:	Everyday<br>For more than 10 consecutive days<br>Before a race<br>Before a training session<br>None of the above
Using anti-inflammatories during exercise is…	Safe<br>A good idea to prevent stiffness and pain<br>Associated with higher risks of side-effects<br>Associated with lower risks of side-effects<br>Performance enhancing
Using pain medication above the recommended dose…	Will speed up recovery<br>Will increase your risk of side-effects<br>Increases the benefits of the medication<br>Prevents further injury<br>Is safe

It is safe to take over-the-counter pain medication if you have been drinking alcohol	True<br>False<br>Don't Know
If the recommended dose of pain medication doesn't relieve your pain, it is safe to take more	True<br>False<br>Don't Know
Local anaesthetic injections can cause heart problems	True<br>False<br>Don't Know
Aspirin can be both an analgesic and an anti-inflammatory	True<br>False<br>Don't Know
Panado® is a stronger pain medication than Codeine	True<br>False<br>Don't Know
Injected corticosteroids (Cortisone) are safer than oral/tablet corticosteroids (Cortisone)	True<br>False<br>Don't Know
It is possible to overdose on Panado® (Paracetamol)	True<br>False<br>Don't Know
Anti-depressant medication can be used to manage pain	True<br>False<br>Don't Know
Paracetamol and anti-inflammatories work in the same way?	True<br>False<br>Don't Know
Topical pain medication (gels and patches) have fewer side effects than other pain forms of pain medication?	True<br>False<br>Don't Know
It is safe to use oral (tablets) and topical anti-inflammatories at the same time?	True<br>False<br>Don't Know
All types of topical pain medication have the same side effects?	True<br>False<br>Don't Know
It is safer to use topical pain medication, rather than oral pain medication, if you are using other types of medication (i.e. diabetic or cholesterol medication)?	True<br>False<br>Don't Know

2) Attitudes Towards Pain Medication

The following questions will assess your Attitudes and Beliefs towards the use of pain medication.

Strongly Disagree	Disagree	Neutral	Agree	Strongly Agree

Pain medication should only be used if prescribed by a Doctor:
Pain medication that is prescribed by a Doctor is safer than medication bought over-the-counter:
I would rather use pain medication that I can get over-the-counter than medication given to me by a Doctor:
It is important to know the recommended doses of any pain medication that you use:
It is important to know the potential side effects of all pain medication that you use:
I would use more than the recommended dose of Paracetamol if I was not experiencing any side effects:
I would use more than the recommended dose of NSAIDs if I was not experiencing any side effects:
I would use topical pain medication (Transact® patches, Deep Heat®) rather than oral pain medication (Paracetamol/anti-inflammatory tablets):
I would use topical pain medication (Transact® patches, Deep Heat®) and oral pain medication (Paracetamol/anti-inflammatory tablets) at the same time;
All types of pain medication have the same side effects:
I would use Paracetamol instead of NSAIDs if I was in pain:
I would use Muscle Relaxants instead of NSAIDs if I was in pain:

Taking pain medication before a run will stop me from feeling pain during the run:
Taking pain medication before a run will stop me from feeling pain or stiffness after the run:
I would use NSAIDs instead of Paracetamol while running:
I would use Muscle Relaxants instead of Paracetamol while running:
I would use NSAIDs instead of Muscle Relaxants while running:
Oral pain medication (Paracetamol/anti-inflammatories) are an important part of my running preparations:
Topical pain medication (Transact® patches, Deep Heat) are an important part of my running preparations:
I would use pain medication for training:<br>If I had pain (an injury):<br>To prevent pain or stiffness:
I would use pain medication for a race:<br>If I had pain (an injury):<br>To prevent pain or stiffness:
I would use pain medication as part of my recovery:
I would use pain medication daily in preparation for a race:
I would only use pain medication when running if I was injured:
I would use more than one type of pain medication at one time:
Using pain medication before training or a race will improve performance:
Using pain medication will speed up recovery:
Oral pain medication (Paracetamol/anti-inflammatories) are an important part of my recovery:
Topical pain medication (Transact® patches, Deep Heat®) are an important part of my recovery:
Using pain medication will prevent injury:
Using pain medication will help injuries to heal quicker:
Using pain medication will stop injuries from becoming worse:
I keep pain medication with me during a run "just in case":
Pain medication has an important role in running:
Pain medication has an important role in recovery:
Runners are educated enough with regards to the effects and side-effects of pain medication:
